# Biological Validation of Self-Reported Unprotected Sex and Comparison of Underreporting Over Two Different Recall Periods Among Female Sex Workers in Benin

**DOI:** 10.1093/ofid/ofz010

**Published:** 2019-01-09

**Authors:** Katia Giguère, Luc Béhanzin, Fernand A Guédou, François A Leblond, Ella Goma-Matsétsé, Djimon M Zannou, Dissou Affolabi, René K Kêkê, Flore Gangbo, Moussa Bachabi, Michel Alary

**Affiliations:** 1 Centre de Recherche du CHU de Québec-Université Laval, Canada; 2 Département de Médecine Sociale et Préventive, Université Laval, Québec, Canada; 3 Dispensaire IST, Centre de Santé Communal de Cotonou 1, Bénin; 4 École Nationale de Formation des Techniciens Supérieurs en Santé Publique et en Surveillance Épidémiologique, Université de Parakou, Bénin; 5 Independent Consultant, Montréal, Canada; 6 Faculté des Sciences de la Santé, Université d’Abomey-Calavi, Cotonou, Bénin; 7 Centre National Hospitalier Universitaire HMK de Cotonou, Bénin; 8 Programme Santé de Lutte Contre le Sida, Cotonou, Bénin; 9 Institut National de Santé Publique du Québec, Canada

**Keywords:** female sex workers, misreporting, prostate-specific antigen, sexual behavior, Y-chromosomal DNA

## Abstract

**Background:**

Self-reported unprotected sex validity is questionable and is thought to decline with longer recall periods. We used biomarkers of semen to validate self-reported unprotected sex and to compare underreporting of unprotected sex between 2 recall periods among female sex workers (FSW).

**Methods:**

At baseline of an early antiretroviral therapy and pre-exposure prophylaxis demonstration study conducted among FSW in Cotonou, Benin, unprotected sex was assessed with retrospective questionnaires, and with vaginal detection of prostate-specific antigen (PSA) and Y-chromosomal deoxyribonucleic acid (Yc-DNA). Underreporting in the last 2 or 14 days was defined as having reported no unprotected sex in the recall period while testing positive for PSA or Yc-DNA, respectively. Log-binomial regression was used to compare underreporting over the 2 recall periods.

**Results:**

Unprotected sex prevalence among 334 participants was 25.8% (50.3%) according to self-report in the last 2 (or 14) days, 32.0% according to PSA, and 44.3% according to Yc-DNA. The proportion of participants underreporting unprotected sex was similar when considering the last 2 (18.9%) or 14 days (21.0%; proportion ratio = 0.90; 95% confidence interval, 0.72–1.13). Among the 107 participants who tested positive for PSA, 19 (17.8%) tested negative for Yc-DNA.

**Conclusions:**

Underreporting of unprotected sex was high among FSW but did not seem to be influenced by the recall period length. Reasons for discrepancies between PSA and Yc-DNA detection, where women tested positive for PSA but negative for Yc-DNA, should be further investigated.

Accurate measurement of unprotected sex is essential in human immunodeficiency virus (HIV)/sexually transmitted infection (STI) surveillance, treatment, and prevention research because misclassification of unprotected sex as an exposure or an outcome might lead to invalid studies. The most affordable and commonly used method to assess unprotected sex is the questionnaire. However, validity of self-report of sexual behaviors is questionable. Among factors that are suspected to alter validity of self-report is recall bias, where longer recall periods and more frequent sexual behaviors are thought to decrease reliability of reported frequency of sexual behaviors [1]. Some studies using daily coital diaries as a gold standard tool to collect sexual behavior data have observed inaccuracies with data collected by retrospective questionnaires among female sex workers (FSW), men who have sex with men, or heterosexual youth and adults [2–8]. Daily coital diary indeed allows prospective collection of data, which reduces problems associated with long-term recall such as forgetting and telescoping [9]. However, data collected by diaries are still self-reported and thus subject to social desirability bias and even to recall bias if not recorded on a daily basis as instructed. An objective assessment of sexual behaviors over different recall periods is required to evaluate the potential impact of longer recall periods on validity of self-reported data.

Previous studies have shown that prostate-specific antigen (PSA) and Y-chromosomal deoxyribonucleic acid (Yc-DNA) are both valid biomarkers of recent semen exposure among women that can be detected up to 2 and 14 days after semen exposure, respectively [10–12]. Studies conducted among FSW, high-risk women, or women from the general population have shown that between 7.7% and 35.5% of all participating women reported no unprotected sex in the last 2 days while testing positive for PSA [13–18]. Other studies conducted among high-risk women or female adolescents have shown that from 8.8% to 19.4% of all study participants reported no unprotected sex in the last 14 days while testing positive for Yc-DNA [19, 20]. To date, no study conducted in observational settings used PSA and Yc-DNA in the same population to assess validity of self-reported unprotected sex.

Concomitant use of PSA and Yc-DNA to assess validity of self-report could allow an objective comparison of underreporting over the last 2 and 14 days. Our objectives were to validate self-report of unprotected sex by the use of PSA and Yc-DNA and to objectively compare underreporting of unprotected sex over the last 2 and 14 days among FSW in Cotonou, Benin.

## METHODS

### Participants

This study uses data that were collected at baseline of a prospective observational demonstration study that aimed to assess feasibility and usefulness of early antiretroviral treatment (E-ART) and pre-exposure prophylaxis (PrEP) among FSW in Cotonou, Benin (NCT02237027). More details on the E-ART/PrEP study are provided elsewhere [21]. In brief, eligible FSW from Cotonou and its inner suburbs were recruited in the E-ART/PrEP study from October 2014 to December 2015 at the Dispensaire IST, a clinic providing confidential clinical services and free HIV/STI treatment to FSW. Human immunodeficiency virus-positive FSW were eligible for E-ART if they were ≥18 years old, HIV-treatment naive, and not infected with HIV-2. Human immunodeficiency virus-negative FSW were eligible for PrEP if they were ≥18 years old, had normal renal and liver functions, had no active hepatitis B, and were not pregnant or breastfeeding. At baseline, 2 trained interviewers assessed socio-demographic characteristics as well as sexual behaviors from the last 2 and 14 days using a standardized face-to-face interview in a private setting at the Dispensaire IST. At the same visit, a physician collected vaginal swabs for PSA and Yc-DNA detection.

Participants provided free and informed written consent before recruitment, but they were not informed of the specific purpose of PSA and Yc-DNA detection until the end of the E-ART/PrEP study (December 2016) to limit information bias. The protocol, including procedures for delayed information, was approved by the Benin National Ethics Committee for Health Research and the ethics committee of CHU de Québec-Université Laval.

### Self-Report of Unprotected Sex

For each of the 2 recall periods (last 2 and last 14 days), participants were asked the following: the number of vaginal sex acts with clients, regular partners, and other types of partners (ie, nonpaying and nonregular); frequency of condom use with these partners (never, less than half of the time, at least half of the time, or always); and whether a condom broke or slipped. Self-report of unprotected sex in the last 2 or 14 days was defined as reporting at least 1 vaginal sex act with any type of partner and inconsistent condom use or condom breakage or slippage in the last 2 or 14 days, respectively.

### Prostate-Specific Antigen and Y-Chromosomal Deoxyribonucleic Acid Detection

After collection by a clinician, vaginal swabs were stored at −20°C for a maximum of 1 week before extraction of PSA and total DNA. For PSA extraction, each vaginal swab was eluted for 2 hours at 4°C in an extraction solution provided in the ABAcard p30 kit (Abacus Diagnostics, West Hills, CA), a commercially available rapid immunodetection test for PSA. After incubation, the swab was pressed gently on the inner wall of the tube to recover the maximum amount of solution, and the extract was centrifuged at 10 000 ×*g* for 5 minutes at room temperature. The supernatant was recovered and stored at −20°C for a maximum of 1 week before PSA detection. Total DNA was extracted from the remaining cellular pellet using the commercially available QIAamp DNA extraction kit (QIAGEN AG, Basel, Switzerland) according to the manufacturer’s instructions. The total DNA extract was stored at −80°C for a maximum of 2 weeks before Yc-DNA detection. We detected PSA by the use of ABAcard p30 according to the manufacturer’s instructions. In brief, we added 200 µL of the vaginal extract solution (supernatant) to the sample well of the strip test and incubated the strip test at room temperature for 10 minutes. A pink line at the control (C) position only indicated a negative result, whereas a pink line at both the test (T) and C positions indicated a positive result. We observed no inconclusive result (i.e., absence of a pink line at the C position).

We detected Yc-DNA by the use of a previously described nested polymerase chain reaction (PCR) assay targeting the testis-specific protein Y-encoded (TSPY) family of homologous genes (K.G., F.A.L., E.G.-M., V.D., L.B., F.A.G., M.A., manuscript submitted). We amplified samples in replicates of 3 with a Bio-Rad iCycler (Bio-Rad Laboratories, Hercules, CA), and we visualized amplifications results under UV transillumination after electrophoresis on a 2% agarose gel and staining with Gel Red (Biotium Inc., Fremont, CA). We concluded to a negative result if none of the replicates had amplification or to a positive result if at least 1 replicate had amplification. We included a no template control (nuclease-free water), a negative control (DNA from peripheral blood mononuclear cells from a female donor), and a positive control (DNA from peripheral blood mononuclear cells from a male donor) in each PCR assay. None of the no template or negative controls had amplification, and all nested PCR products (positive controls and positive tests) were of expected size. All laboratory procedures were conducted by female technicians to avoid male DNA contamination.

### Underreporting of Unprotected Sex

Underreporting in the last 2 days was defined as having reported no unprotected sex in the last 2 days while testing positive for PSA, whereas underreporting in the last 14 days was defined as having reported no unprotected sex in the last 14 days while testing positive for Yc-DNA. We calculated underreporting among women who reported no unprotected sex and among the total study population.

### Statistical Analysis

At baseline, we assessed unprotected sex with 4 methods: self-report of unprotected sex in the last 2 days, self-report of unprotected sex in the last 14 days, a rapid PSA detection test, and a nested PCR targeting Yc-DNA. We restricted analyses to participants who had complete data for each of the 4 methods. To assess and compare unprotected sex prevalence estimates according to self-report in the last 2 days (14 days) or to PSA (Yc-DNA) detection, we simultaneously fit a model (1 model per recall period) for the 2 outcomes using a log-binomial regression [22]. A generalized estimating equation (GEE) with a log link function, a compound symmetry working correlation matrix, and a binomial distribution was applied to account for the dependance between unprotected sex measures from the same participant.

We also assessed and compared the prevalence of underreporting in the last 2 days and in the last 14 days in the total study population. We simultaneously fit a log-binomial model for the 2 underreporting measures and applied GEE as described for unprotected sex. We chose to compare underreporting in the total study population, and not among women who reported no unprotected sex, because restricting the analysis to women who reported no unprotected sex would have blocked an important causal path between the recall period length and underreporting (Supplementary Figure 1). Moreover, carrying out this analysis in the total study population allowed us to compare identical groups of women (i.e., same women), which prevented potential confounding bias.

We tested all comparisons by contrasts (χ^2^ tests). In addition, we tested agreement of results between the different unprotected sex measures using the McNemar’s test. Statistical analyses were conducted using SAS Studio, version 3.7.1 (SAS Institute Inc., Cary, NC).

## RESULTS

### Study Population

Of the 361 FSW that were recruited in the E-ART/PrEP study, 334 (92.5%) had complete data for the 4 unprotected sex measures at recruitment and were included in this analysis. Mean age was 33.4 years (standard deviation = 9.3). Most participants were Beninese (50.9%), had less than a secondary education (69.9%), were not married (98.2%), used no hormonal contraception (86.2%), had already attended a condom use demonstration (94.0%), identified the condom as an effective means to protect against HIV (98.2%), and perceived themselves at risk for HIV infection (88.5%). Participants’ baseline characteristics are presented in Table 1, and the distribution of the number of vaginal sex acts in the last 2 and 14 days with clients and regular partners are presented in Supplementary Figure 2.

**Table 1.** Baseline Characteristics of Participating Female Sex Workers (n = 334)

Characteristics	n (%) or Mean (SD)
Mean age (SD), years	33.4 (9.3)
Country of Origin	
Benin	171 (51.2)
Togo	84 (25.1)
Nigeria	53 (15.9)
Ghana	22 (6.6)
Cameroon	2 (0.6)
Congo	1 (0.3)
Mali	1 (0.3)
Education (n = 332)	
None	105 (31.6)
Primary	127 (38.3)
Secondary	91 (27.5)
University	8 (2.4)
Marital Status	
Never married	114 (34.2)
Divorced/separated	157 (47.2)
Widowed	56 (16.8)
Married	6 (1.8)
Contraceptive Method	
None	215 (64.6)
Condom only	57 (17.1)
Hormonal	46 (13.8)
Intrauterine device	3 (0.9)
Menopause	3 (0.9)
Traditionnal methods	9 (2.4)
Alcohol use in the last 4 days (n = 327)	161 (49.2)
Drug use in the last 3 months^a^ (n = 244)	21 (8.6)
Already had an STI	173 (52.0)
Already attended a condom use demonstration	313 (94.0)
Identified the condom as an effective mean to protect against HIV	327 (98.2)
Perceive a risk of HIV infection (n = 330)	292 (88.5)
HIV positive	96 (28.7)
Sexual Behaviors in the Last 2 Days	
At least 1 vaginal douche (n = 201)^b^	201 (100.0)
Had sex with clients	254 (76.5)
Mean number of vaginal sex acts with clients (SD)	4.1 (4.6)
Had sex with a regular partner	65 (19.6)
Mean number of vaginal sex acts with a regular partner (SD)	0.3 (0.6)
Had sex with other type of partner^c^	3 (0.9)
Sexual Behaviors in the Last 14 Days	
Had sex with clients	301 (91.5)
Mean number of vaginal sex acts with clients (n = 330) (SD)	19.3 (23.0)
Had sex with a regular partner	129 (38.9)
Mean number of vaginal sex acts with a regular partner (SD)	0.9 (1.4)
Had sex with other type of partner^c^	9 (2.7)

Abbreviations: HIV, human immunodeficiency virus; SD, standard deviation; STI, sexually transmitted infection.

^a^The only drug used was marijuana. Missing data (n = 90) because of nonresponse.

^b^The first 160 participants have missing data for vaginal douching because of the late introduction of questions on vaginal douching practices after the beginning of the recruitment.

^c^Other type of partner = nonpaying and nonregular partner.

### Self-Report of Unprotected Sex Validity and Underreporting of Unprotected Sex

Table 2 shows the comparison between self-reported unprotected sex in the last 2 days and PSA detection results. Among 334 FSW, 26.0% reported having had unprotected sex in the last 2 days, whereas 32.0% tested positive for PSA (proportion ratio [PR] = 0.81; 95% confidence interval [CI], 0.66–1.00). McNemar’s test between self-report in the last 2 days and PSA was borderline significant (*P* = .065). More than one quarter (25.5%) of FSW reporting no unprotected sex in the last 2 days (n = 247) tested positive for PSA.

**Table 2.** Agreement Between Self-Reported Unprotected Sex in the Last 2 Days and PSA at Baseline of an E-ART and PrEP Demonstration Study Conducted Among Female Sex Workers in Cotonou, Benin

	Self-Report of Unprotected Sex in the Last 2 Days			
PSA	Yes	No	Total	*P* Value^a^
Positive	44	63	107 (32.0%)	.065
Negative	43	184	227	
Total	87 (26.0%)	247	334	

Abbreviations: E-ART, early antiretroviral treatment; PrEP, pre-exposure prophylaxis; PSA, prostate-specific antigen.

^a^McNemar’s test.

In the last 14 days (Table 3), 50.6% of FSW reported having had unprotected sex and 44.3% tested positive for Yc-DNA (PR = 1.14; 95% CI, 0.98–1.34). We observed no statistically significant difference between self-report in the last 14 days and Yc-DNA (*P* = .115, McNemar’s test). Among FSW who reported no unprotected sex in the last 14 days (n = 165), 70 (42.4%) tested positive for Yc-DNA.

**Table 3.** Agreement Between Self-Reported Unprotected Sex in the Last 14 Days and Yc-DNA at Baseline of an E-ART and PrEP Demonstration Study Conducted Among Female Sex Workers in Cotonou, Benin

	Self-Report of Unprotected Sex in the Last 14 Days			
Yc-DNA	Yes	No	Total	*P* Value^a^
Positive	78	70	148 (44.3%)	.115
Negative	91	95	186	
Total	169 (50.6%)	165	334	

Abbreviations: E-ART, early antiretroviral treatment; PrEP, pre-exposure prophylaxis; Yc-DNA, Y-chromosomal deoxyribonucleic acid.

^a^McNemar’s test.

In the total study population (n = 334), 18.9% of the FSW underreported unprotected sex in the last 2 days and 21.0% did so in the last 14 days. We observed no statistically significant difference between the proportion of women underreporting unprotected sex over the last 2 days and 14 days in the total study population (PR = 0.90; 95% CI, 0.72–1.13).

### Joint Distribution of Prostate-Specific Antigen and Y-Chromosomal Deoxyribonucleic Acid Results

Table 4 shows the joint distribution of PSA and Yc-DNA results. Of all included participants, 50.0% tested negative for both biomarkers, 26.3% tested positive for both biomarkers, and 18.0% tested negative for PSA but positive for Yc-DNA, and 5.7% tested positive for PSA but negative for Yc-DNA. Among the 107 participants who tested positive for PSA, 19 (17.8%) tested negative for Yc-DNA. Participants who tested positive for PSA but negative for Yc-DNA tended to report unprotected sex in the last 2 days more often (9 of 19 = 47.7%) than women who tested positive for both biomarkers (35 of 88 = 39.8%). However, the difference was not statistically different (*P* = .54).

**Table 4.** Joint Distribution of PSA and Yc-DNA Results at Baseline of an E-ART and PrEP Demonstration Study Conducted Among Female Sex Workers in Cotonou, Benin

	PSA		
Yc-DNA	Positive	Negative	Total
Positive	88	60	148 (44.3%)
Negative	19	167	186
Total	107 (32.0%)	227	334

Abbreviations: E-ART, early antiretroviral treatment; PrEP, pre-exposure prophylaxis; PSA, prostate-specific antigen; Yc-DNA, Y-chromosomal deoxyribonucleic acid.

## DISCUSSION

This study was undertaken to validate self-report of unprotected sex by the use of PSA and Yc-DNA and to objectively compare underreporting of unprotected sex over the last 2 and 14 days among FSW in Cotonou, Benin. Although we observed no statistically significant difference between the proportion of FSW reporting unprotected sex in the last 2 days (or 14 days) and the proportion of FSW testing positive for PSA (or Yc-DNA), our results suggest lack of validity of self-report of unprotected sex. Indeed, among women who reported no unprotected sex in the last 2 or 14 days, 25.5% and 42.4% tested positive for PSA or Yc-DNA, respectively. In the total study population, the proportions of women who underreported unprotected sex in the last 2 or 14 days were 18.9% and 21.0%, respectively. Those results are consistent with previous studies that all observed underreporting in the last 2 or 14 days using PSA or Yc-DNA to validate self-report among FSW, high-risk women, women from the general population, or female adolescents [13–18].

Several factors might account for underreporting in our population. The first one, which is also the most likely, is social desirability. Indeed, participants were provided free condoms, were instructed to consistently use them, knew that condoms protect against HIV, and perceived themselves at risk to get HIV. In this context, participants might have feared being judged if they admitted not having used condoms [23]. Second, unawareness of condom breakage or slippage might also have led to underreporting. Indeed, PSA and Yc-DNA were shown to be sensitive enough to detect low amount of semen exposure similar to the magnitude of an exposure from a condom malfunction [10–12, 19, 24]. Third, misunderstanding of questions by participants might have led them to underreport. Fourth, recall bias might also have accounted for underreporting. Previous studies have shown that validity of self-reported sexual behaviors decreases with more frequent behaviors and with longer recall periods [1, 4]. In our study, the number of sexual encounters was high, especially with clients, both in the last 2 and 14 days. Participants might have had difficulties to properly evaluate when unprotected sex events took place in the specified recall periods, especially for more distant events due to high rates of sexual encounters.

Our study is the first to use both PSA and Yc-DNA in the same population in observational settings to validate self-report of unprotected sex, which allowed us to objectively compare underreporting over 2 different recall periods. We observed a similar proportion of participants underreporting unprotected sex in both the last 2 and 14 days. The small difference in length between the 2 recall periods could explain that absence of difference. Indeed, participants might have had the same ability to remember events from the last 2 days or from the last 14 days because both recall periods were relatively short. However, this result must be interpreted cautiously because some of our results suggest that the relative performance of both biomarkers to detect unprotected sex over their respective recall periods might have not been equivalent.

Indeed, because PSA can be detected over a shorter time period than Yc-DNA after semen exposure, a woman testing positive for PSA should also test positive for Yc-DNA. Noticeably, in our study, 19 (17.8%) of the participants testing positive for PSA actually tested negative for Yc-DNA. One explanation for this inconsistency could be that PSA yielded false-positive results. A controlled laboratory experiment has previously shown that a commercial lubricant (K-Y Brand Jelly, Johnson & Johnson) and a spermicide (Gynol II, Ortho) can induce false-positive results with ABAcard p30 [25]. We did not assess use of lubricants and spermicides in our study. However, lubricant use was assessed in a sociobehavioral study that was conducted in 2013 among 450 FSW from 9 cities in Benin, including Cotonou (F.K., F.G., M.A.-G., L.B., G.B., M.A., unpublished observations, 2018). In this study, 64.0% of FSW reported using lubricants, 27.6% of whom reported using K-Y gel. Thus, it is possible that some of our participants used K-Y gel over the course of our study, which could have induced false-positive PSA results.

Another likely explanation to the 19 women who tested positive for PSA but tested negative for Yc-DNA is that Yc-DNA yielded false-negative results. Jamshidi et al [12] have previously observed that, despite a shorter time decay, PSA was detected more often than Yc-DNA from 0 to 6 hours after semen exposure. That is, a woman who had very recent unprotected sex could potentially test positive for PSA but negative for Yc-DNA. In our study, women who tested positive for PSA but negative for Yc-DNA tended to report unprotected sex in the last 2 days (47.7%) more often than women who tested positive for both biomarkers (39.8%), although this difference was not statistically significant (*P* = .54). If women who reported unprotected sex in the last 2 days actually had sex in the previous few hours, it is possible that they had a false-negative Yc-DNA result while testing correctly positive for PSA. Jamshidi et al [12] also observed that Yc-DNA results had more inconsistencies than PSA results, which could be explained by different “hit or miss” probabilities of the swab to encounter sperm containing Yc-DNA or seminal fluid containing PSA, and this could lead to a higher rate of false-negative Yc-DNA than PSA results.

If false-positive PSA and/or false-negative Yc-DNA results occured, PSA might have overestimated unprotected sex and underreporting in the last 2 days, and/or Yc-DNA might have underestimated unprotected sex and underreporting in the last 14 days. If so, the misclassification of underreporting over the 2 recall periods might have impaired our capacity to observe an association between the recall period length and underreporting.

This study has some limitations. First, the relatively low sample size has led to wide 95% CI that could have impaired our capacity to detect significant differences between self-report of unprotected sex and biomarker detection. Second, PSA and Yc-DNA concentrations decline rapidly after semen exposure, and only a minority of women will test positive for PSA or Yc-DNA until 2 and 14 days, respectively. In previous studies, only 21% to 69% of women tested positive for PSA 24 hours after semen exposure and only 12% to 64% tested positive for Yc-DNA 6 to 7 days after semen exposure [10, 12, 26]. Moreover, in our study, high prevalence of vaginal douching is hypothesized to have accelerated semen decay from the vaginal vault, which could have affected semen detection. Low concentration of semen was also previously observed among samples from the same population (K.G., F.A.L., E.G.-M., V.D., L.B., F.A.G., M.A., manuscript submitted). That is, prevalences of unprotected sex as measured by PSA and Yc-DNA are likely to be underestimated, and, by extension, underreporting is also likely to have been underestimated in our study. This also prevented us from assessing overreporting of unprotected sex because participants overreporting unprotected sex cannot be distinguished from those who accurately report unprotected sex but test negative for biomarkers due to the clearance time of the latter. Third, our results might not be generalizable to other populations. Indeed, underreporting is associated with factors such as education, chlamydia, and the number of sexual partners [14, 15, 27], all of which might have different prevalence among FSW compared to, for example, women from the general population or female adolescents.

## CONCLUSIONS

In conclusion, the high prevalence of underreporting of unprotected sex among FSW from Cotonou is of concern and suggests that self-reported data should be interpreted cautiously. Where possible, unprotected sex should be assessed using both self-report and biomarkers of recent semen exposure. Underreporting of unprotected sex did not seem to be influenced by the recall period length; however, discrepancies between PSA and Yc-DNA results, where women tested positive for PSA but negative for Yc-DNA, suggest that the relative performance of both biomarkers to detect unprotected sex over their respective recall periods might not have been equivalent. Reasons for these discrepancies should be further investigated.

## Supplementary Data

Supplementary materials are available at *Open Forum Infectious Diseases* online. Consisting of data provided by the authors to benefit the reader, the posted materials are not copyedited and are the sole responsibility of the authors, so questions or comments should be addressed to the corresponding author.

ofz010_suppl_supplementary_figure_1

ofz010_suppl_supplementary_figure_2
