# Supplementary material for: Biological Validation of Self-Reported Unprotected Sex and Comparison of Underreporting Over Two Different Recall Periods Among Female Sex Workers in Benin
Source: Open Forum Infect Dis. 2019 Jan 9;6(2):ofz010. doi: 10.1093/ofid/ofz010 (PMC6364862; doi:10.1093/ofid/ofz010)
Supplement: ofz010_suppl_supplementary_figure_1 [file ofz010_suppl_supplementary_figure_1.docx]

**Supplementary Figure 1—Directed acyclic graph of the causal paths between recall period length and under-reporting of unprotected sex**

We chose to compare under-reporting in the total study population, and not among women who reported no unprotected sex, because restricting analysis to women who reported no unprotected sex (adjusting for self-report of unprotected sex) would have blocked an important causal path between the recall period length and under-reporting.
