# Supplementary material for: Biological Validation of Self-Reported Unprotected Sex and Comparison of Underreporting Over Two Different Recall Periods Among Female Sex Workers in Benin
Source: Open Forum Infect Dis. 2019 Jan 9;6(2):ofz010. doi: 10.1093/ofid/ofz010 (PMC6364862; doi:10.1093/ofid/ofz010)
Supplement: ofz010_suppl_supplementary_figure_2 [file ofz010_suppl_supplementary_figure_2.docx]

**Supplementary Figure 2—Distribution of the number of vaginal sex acts at baseline of an E-ART and PrEP demonstration study conducted among female sex workers in Cotonou, Benin** A) Distribution of the number of vaginal sex acts with clients in the last two days; B) Distribution of the number of vaginal sex acts with clients in the last 14 days; C) Distribution of the number of vaginal sex acts with a regular partner in the last two days; D) Distribution of the number of vaginal sex acts with a regular partner in the last 14 days.
